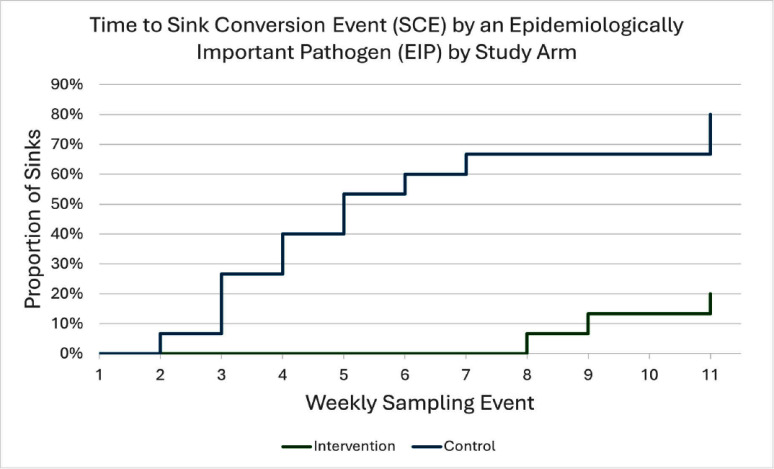# Efficacy of a Disinfectant in Reducing Pathogen Contamination in Renovated Inpatient In-Room Sinks: A Randomized Controlled Trial

**DOI:** 10.1017/ash.2025.221

**Published:** 2025-09-24

**Authors:** Bobby Warren, Amanda Graves, Guerbine Fils-aime, Aaron Barrett, Isadora Mamikunian, Becky Smith, Deverick Anderson

**Affiliations:** 1 Duke Center for Antimicrobial Stewardship and Infection Prevention; 2 Duke University Hospital; 3 Undergraduate Researcher; 4 Duke University Medical Center

## Abstract

**Introduction:** The prevention of colonization of new inpatient room sinks with epidemiologically important pathogen (EIP) is poorly understood. **Methods:** We performed a randomized controlled trial and microbiological analysis to describe the timing and frequency of EIP contamination of in-room handwashing sinks in a renovated general medicine unit and to test the efficacy of a disinfectant on wastewater drains. Unit renovations included new distal plumbing for all unit sinks. Sinks were randomized 1:1 to intervention and control. 15 intervention sinks underwent the application of a foamed disinfectant into each study sink’s drain (Virasept [Ecolab]) every Monday, Wednesday, and Friday. 15 control sinks underwent standard disinfection. Microbiological samples were taken every week from 3 locations from each study sink: the top of the bowl, the tail pipe, and the p-trap. The primary outcome was sink conversion events (SCEs), defined as species-specific-EIP contamination of a sink in which that EIP had not previously been detected. EIPs were defined as Enterobacterales, Acinetobacter spp., Pseudomonas aeruginosa, and Stenotrophomonas maltophilia. Sink hygiene compliance was also measured. **Results:** Sink samples were obtained 11 times from July 2024 to October 2024 giving 990 total cultures, 495 in each study arm. The first sampling occurred after renovations were completed and one week before patients returned to the unit. In total, 314 patients were admitted to the study unit during the study period. We observed 15 SCEs (68%) overall; 3 sinks (20%) in the intervention and 12 (80%) in the control arms (p < 0 .01) (Figure 1). Overall, 135 EIPs were recovered: 27 from intervention and 108 from control sinks (p < 0 .01). On the first sampling, 6 intervention and 3 control sinks already harbored EIP. Of those, all 6 in the intervention arm were not detected in subsequent samplings whereas all 3 in the control arm were. The [DAM1] [A2] most common organisms recovered were Acinetobacter spp. (70 [52%]), Stenotrophomonas maltophilia (29 [21%]), and Enterobacter spp. (23 [17%]). Of the 300 opportunities to measure sink hygiene compliance, 229 (76%) did not meet compliance, most commonly containing absorbent pads. **Discussion:** In a renovated general medicine unit, the application of the intervention led to significantly lower SCEs (20% vs. 80%) and EIPs (27 vs. 108) recovered from study stinks compared to control. Sink hygiene compliance was suboptimal, with 76% of observed instances involving inappropriate sink use. These findings highlight the importance of effective sink disinfection and sink hygiene protocols in reducing the risk of pathogen transmission.

**Figure:**